# Ocean Acidification Impairs Foraging Behavior by Interfering With Olfactory Neural Signal Transduction in Black Sea Bream, *Acanthopagrus schlegelii*

**DOI:** 10.3389/fphys.2018.01592

**Published:** 2018-11-20

**Authors:** Rong Jiahuan, Su Wenhao, Guan Xiaofan, Shi Wei, Zha Shanjie, He Maolong, Wang Haifeng, Liu Guangxu

**Affiliations:** ^1^College of Animal Science, Zhejiang University, Hangzhou, China; ^2^Lucta (Guangzhou) Flavours Co., Ltd., Guangzhou, China

**Keywords:** ocean acidification, black sea bream, foraging behavior, olfactory transduction, neurotransmitters

## Abstract

In recent years, ocean acidification (OA) caused by oceanic absorption of anthropogenic carbon dioxide (CO_2_) has drawn worldwide concern over its physiological and ecological effects on marine organisms. However, the behavioral impacts of OA and especially the underlying physiological mechanisms causing these impacts are still poorly understood in marine species. Therefore, in the present study, the effects of elevated *p*CO_2_ on foraging behavior, *in vivo* contents of two important neurotransmitters, and the expression of genes encoding key modulatory enzymes from the olfactory transduction pathway were investigated in the larval black sea bream. The results showed that larval sea breams (length of 4.71 ± 0.45 cm) reared in *p*CO_2_ acidified seawater (pH at 7.8 and 7.4) for 15 days tend to stall longer at their acclimated zone and swim with a significant slower velocity in a more zigzag manner toward food source, thereby taking twice the amount of time than control (pH at 8.1) to reach the food source. These findings indicate that the foraging behavior of the sea bream was significantly impaired by ocean acidification. In addition, compared to a control, significant reductions in the *in vivo* contents of γ-aminobutyric acid (GABA) and Acetylcholine (ACh) were detected in ocean acidification-treated sea breams. Furthermore, in the acidified experiment groups, the expression of genes encoding positive regulators, the olfaction-specific G protein (*Golf*) and the G-protein signaling 2 (*RGS2*) and negative regulators, the G protein-coupled receptor kinase (*GRK*) and *arrestin* in the olfactory transduction pathway were found to be significantly suppressed and up-regulated, respectively. Changes in neurotransmitter content and expression of olfactory transduction related genes indicate a significant disruptive effect caused by OA on olfactory neural signal transduction, which might reveal the underlying cause of the hampered foraging behavior.

## Introduction

Since the industrial revolution, anthropogenic activities such as cement production and the utilization of fossil fuels have released large amounts of carbon dioxide (CO_2_) into the atmosphere, resulting in a substantial rise of atmospheric CO_2_ partial pressure (*p*CO_2_) (Widdicombe and Spicer, 2008). According to the predictions of the Intergovernmental Panel on Climate Change (IPCC), the atmospheric CO_2_ concentration has increased from a pre-industrial level of approximately 280 ppm to a present level of approximately 400 ppm (Team et al., 2014). An enormous amount of anthropogenic CO_2_ (approximately 1/4 to 1/3) has been absorbed by the ocean, and while this mitigates the growth of atmospheric CO_2_ concentration, it leads to ocean acidification (OA), the decrease in the pH of surface seawater induced by elevated *p*CO_2_ (Caldeira and Wickett, 2003; Sabine et al., 2004). Compared to pre-industrial levels, the current pH of surface seawater has decreased by 0.1 units and is predicted to drop by another 0.3 to 0.4 units by the end of 21st century and 0.7 to 0.8 units by the year of 2300 (Kleypas et al., 1999; Sabine et al., 2004; Orr et al., 2005).

In recent years, OA has drawn worldwide concern over its physiological and ecological effects on marine organisms, and it has been shown that a series of physiological processes, such as fertilization (Parker et al., 2009; Sewell et al., 2013; Shi et al., 2017a,b), embryonic development (Egilsdottir et al., 2009; Przeslawski et al., 2015; Gravinese, 2017), metabolism (Lannig et al., 2010; Carter et al., 2013; Kroeker et al., 2013; Wittmann and Pörtner, 2013; Zhao et al., 2017a,b), immunity (Bibby et al., 2008; Liu et al., 2016; Wang et al., 2016; Su et al., 2017, 2018), and behavior (Clements and Hunt, 2015; Nagelkerken and Munday, 2016; Peng et al., 2017), in various marine species can be affected by ocean acidification. In addition to altering pH, OA also reduces the availability of carbonate ions by decreasing the saturation state of calcium carbonate (CaCO_3_) (Kleypas et al., 1999). Therefore, most studies investigating the physiological effects of OA have been conducted with calcifying organisms such as corals, echinoderms, and bivalve mollusks (Fine and Tchernov, 2007; Havenhand et al., 2008; Gazeau et al., 2010; Reuter et al., 2011; Hofmann and Bischof, 2014; Bhadury, 2015; Segman et al., 2016). However, the effects of OA on other marine species such as fishes remain poorly understood to date.

In recent years, a series of studies have demonstrated that OA can impair the olfactory functions of a range of marine fish species (Munday et al., 2009, 2010, 2013; Dixson et al., 2010, 2015; Cripps et al., 2011; Ferrari et al., 2011; Devine et al., 2012; McMahon et al., 2018). Since a robust olfactory sense is essential for the survival of marine fishes to avoid predators, to look for suitable habitats, and to locate food sources, olfactory impairment caused by OA has profound impacts on marine fish species (Munday et al., 2009; Gardiner et al., 2014; Yopak et al., 2015). Furthermore, it has been suggested that the olfactory and behavioral impairments induced by elevated *p*CO_2_ levels are probably due to alterations in the function of neurotransmitters, which play a crucial role in the transduction of olfactory neural signals (Nilsson et al., 2012; Roggatz et al., 2016; Clements et al., 2017). In order to maintain an acid-base balance, marine species including fish and invertebrates accumulate HCO_3_^-^ with compensatory reduction in Cl^-^ in their plasma and tissues, which changes the transmembrane gradients of these anions and subsequently excites the GABA receptors (Baker et al., 2009; Esbaugh et al., 2012; Nilsson et al., 2012; Heuer and Grosell, 2014; Heuer et al., 2016). Since the abnormal olfactory preferences of fish can be rescued by gabazine, an antagonist of GABA receptor, the abnormal behavior under OA scenarios may partially attribute to the over-excitation of GABA receptors (Nilsson et al., 2012; Lai et al., 2015). Theoretically, since it is the binding of neurotransmitters with the corresponding receptors that trigger downstream behavioral and physiological regulations, changes in *in vivo* contents of neurotransmitters may offset or reinforce the impacts caused by alterations in their receptors. However, to the best of our knowledge, the direct impact of OA on the *in vivo* contents of neurotransmitters has yet to be investigated. Moreover, to generate olfactory neural signals, odor cues need to bind to corresponding receptors to trigger a cascade of cellular signaling events (Kaupp, 2010; Leduc et al., 2013). However, it remains unknown whether key molecules from the olfactory transduction cascade pathway will be affected by elevated *p*CO_2_. These paucities have put significant constrains to a better understanding on the physiological mechanisms underlying the behavioral impairment under OA scenarios.

Black sea bream, *Acanthopagrus schlegelii*, a euryhaline, omnivorous fish, is one of the major commercial fish species in the Asian Pacific (Ji et al., 2003; Ma et al., 2008). Well developed breeding and culture techniques make black sea bream a tractable fish species to address general questions such as the physiological mechanism causing behavioral impairments (Nip et al., 2003). In addition, it has been shown that seasonal variation and uneven food distribution in the ocean often causes periodic food insufficiency for black sea bream (Nip et al., 2003). Since olfaction is an essential physiological mechanism triggering foraging behavior, any disruption brought by environmental changes, such as ocean acidification, could subject black sea bream to malnutrition and subsequently lead to reduction in growth and survivor rates, which may further aggravate the decline of natural population caused by overfishing. Since little is known about the behavioral impacts and the physiological mechanisms behind the behavioral impairment of fish species under OA scenarios to date, the present study was conducted to determine whether the foraging behavior, in terms of the efficiency to detect olfactory cue of food, will be affected by near-future ocean acidification. More importantly we saught to explore whether the behavioral change detected, if any, may be due to an interfered olfactory signal transduction as reflected by the *in vivo* contents of neurotransmitters (GABA and ACh) and the expression of key genes modulating olfactory signal transduction.

## Materials and Methods

### Ethics Statement

This study is performed in accordance with the Animal Ethics Committee in the School of Medicine, Zhejiang University (ETHICS CODE Permit NO. ZJU2011-1-11-009Y, issued by the Animal Ethics Committee in the School of Medicine, Zhejiang University).

### Experimental Animals and Acclimation

Since larvae are generally more susceptible to environmental changes and the recruitment of fish population can be significantly affected by their nutrition condition (Ishimatsu et al., 2004; Murphy et al., 2014), larval black sea breams were investigated in the present study. Larvae of regular size (length of 4.71 ± 0.45 cm, weight at 2.59 ± 0.71 g) from one spawning event using multiple parents were purchased from the Dongtou fish-breeding farm. The hatchery and rearing of these larvae were conducted in the open sea net cages with seawater at the ambient pH (∼8.10). Larvae sea breams were immediately transferred to the Qingjiang Station of Zhejiang Mariculture Research Institute, Wenzhou, China, in June 2017 and were acclimated for a week in a 500 L tank filled with 350 L of aerated, flowing seawater (temperature at 23.71 ± 0.08°C, pH at 8.10 ± 0.01, and salinity at 20.74 ± 0.01) before the experiment. During the acclimation period, black sea breams were fed with commercial pellet feed (diameter of 1.5 mm) twice daily at 9 AM and 5 PM. After the acclimation, healthy individuals without physical injury were used for the experiments.

### Ocean Acidification Treatment and Seawater Chemistry Monitoring

According to the near-future OA scenarios predicted by the IPCC, pH levels of 8.1, 7.8, and 7.4 were employed to simulate the pH levels at present and in the years 2100 and 2250, respectively. According to the method of Zhao et al. (2017a), the stimulation of the acidified scenario was achieved by bubbling dry air or a mixture of carbon dioxide and dry air with different but constant percentages. Once the pH of each experimental tank reached equilibrium at corresponding desired pH through aeration, 90 sea bream individuals were randomly selected from the acclimation tank and equally assigned into 9 (3 treatments × 3 replicates) experimental tanks (total volume of 50 L) each containing 30 L of still seawater, pre-adjusted to the corresponding experimental pH values. The exposure was conducted in an air-conditioned indoor laboratory (temperature was set at 24°C) with an exposure time of 15 days and the sea breams were fed with commercial pellet food at satiation rate twice daily (at 9 AM and 5 PM). An hour after feeding, seawater in each experimental tank was replaced with seawater pre-prepared at the corresponding experimental pH values. During the experiment, seawater of each tank was continuously aerated with the corresponding dry air or CO_2_-air mixture to maintain the stability of seawater carbonate chemistry.

To ensure that the chemical parameters of seawater in each tank were consistent throughout the entire experiment, pH, salinity, and temperature were monitored daily and total alkalinity (TA) was determined once a week (Table 1). The pH_NBS_ of each trial was measured by a pH meter (PB-10, Sartorius) and calibrated with NBS standard buffers. Salinity was measured with a conductivity meter (Multi 3410, WTW) and a mercury thermometer gaged temperature. TA was determined using potentiometric titration (Anderson and Robinson, 1946) with an automatic titrator system (SMTitrino 702, Metrohm). Carbonate system parameters were calculated from the measured pH_NBS_, salinity, temperature, and TA values using the open-source program CO2SYS (Pierrot et al., 2006), with the constants supplied by Mehrbach et al. (1939) and refitted by Dickson and Millero (1987) and the KSO_4_ dissociation constant from Dixson et al. (2010).

**Table 1:** Seawater chemical parameters during the 15-day incubation experiment for the control and *p*CO_2_ acidified groups (mean ± SE).

Target pH	T(°C)	Sal(‰)	pH_NBS_	TA	pCO_2_	DIC	Ωara	Ωcal
						
				(μmol/kg)	(μatm)	(mmol/kg)		
pH 8.1	23.71 ± 0.08	20.74 ± 0.01	8.10 ± 0.01	2056.38 ± 1.63	356.84 ± 4.39	1879.78 ± 6.22	2.24 ± 0.08	3.36 ± 0.13
pH 7.8	23.77 ± 0.09	20.61 ± 0.01	7.80 ± 0.01	2060.37 ± 2.82	776.96 ± 8.22	1984.42 ± 4.75	1.21 ± 0.04	1.97 ± 0.07
pH 7.4	23.78 ± 0.09	20.73 ± 0.01	7.40 ± 0.01	2066.82 ± 3.00	2051.47 ± 21.84	2094.03 ± 3.80	0.51 ± 0.02	0.83 ± 0.03


*Partial pressure of CO_2_, dissolved inorganic carbon, and saturation state of aragonite and calcite were calculated from measured pH_*NBS*_, salinity, temperature, and TA values using the open-source program CO2SYS. T, temperature; Sal, salinity; TA, total alkalinity; *p*CO_2_, CO_2_ partial pressure; DIC, dissolved inorganic carbon; Ωara, aragonite saturation state; Ωcal, calcite saturation state.*

### Foraging Behavior Experiments and Video Analysis

Foraging behavior experiments were performed following published methods with modifications (Ferrari et al., 2012; Dodd et al., 2015; Pistevos et al., 2015). After exposure to corresponding *p*CO_2_ levels for 15 days and then food deprivation for 24 h, 5 black sea bream individuals were randomly selected from each experimental tank and transferred to one end of a white plastic tray (105 × 80 × 30 cm) containing 100 L of sand-filtered still seawater. Since it has been suggested that the behavioral effects of OA will last in fish individuals for 1 or 2 days and the pH of testing sea water will not affect the behavioral responses observed (Munday et al., 2010, 2016), seawater at ambient pH 8.1 was used for the behavioral experiment in the present study. The 5 sea bream individuals were tested simultaneously as one replicate in the analysis. Individuals were allowed to acclimate for 30 s before the introduction of commercial food pellets (50.10 ± 0.81 g), placed approximately 100 cm away from the acclimation area, at the other end of the tray. The food pellets were held in a glass petri dish with an opaque white cover with holes on the top, a design that allows the dispersal of olfactory cues but prevents visual detection of the food. The plastic baffle plate, which was used to restrain the fish in the acclimation area before the assay, was removed and video data of the foraging behavior was collected with an HD digital video camera (T90, Aigo^®^, China) 3 min after the food was introduced. Three replicates were performed for each experimental group and fish tested were discarded to ensure that each individual was only tested once. The curvilinear swimming velocity (VCL) used to approach food source, the linearity (LIN) and wobble (WOB) of fish swimming path, and the time taken to leave the acclimation area and reach the food were determined using the open-source software ImageJ (National Institutes of Health, Bethesda, Maryland, United States) following the method described by Wilsonleedy and Ingermann (2007) and Shi et al. (2017a).

### Content Estimation of Neurotransmitters

After 15-day exposure to corresponding *p*CO_2_ levels, 6 individuals were randomly selected from each experimental treatment tank and dissected on ice. Brain tissue of each individual was carefully removed and used for the determination of *in vivo* GABA and ACh contents using commercial ELISA kits (MLBIO biotechnology Co., Ltd., Shanghai, China) following the manufacturer’s instructions. After weighing, samples were homogenized in ice-cold PBS (0.01 M, pH 7.4) followed by centrifugation at 2000 rpm for 20 min at 4°C. Twenty microliter of the supernatant was mixed with 80 μL working reagent containing a chromogenic reagent. After incubation at room temperature for 20 min, the absorption values at 450 nm were measured with a microplate reader (Thermo Multiskan Go, United States). The contents of GABA and ACh were subsequently determined using the corresponding standard curves.

### Expressions of Key Genes From Olfactory Transduction Pathway

At the end of 15 days of *p*CO_2_ exposure, 6 individuals were randomly selected from each treatment tank and dissected on ice. The brain tissue of each individual was carefully removed and immediately frozen in liquid nitrogen. Total RNA was extracted from the tissue within two weeks of sampling using EASY spin Plus tissues/cells rapid RNA extraction kit (Aidlab, RN2802) following the method described (Peng et al., 2016). The quality and concentration of total RNA obtained were checked by gel electrophoresis and NanoDrop 1000 UV/visible spectrophotometer (Thermo Scientific), respectively. High-quality RNA samples were then reversely transcribed into first strand cDNA immediately using a M-MLV First Strand Kit (Invitrogen, C28025-032) following the manufacturer’s protocols. Fresh cDNA samples or those stored in -20°C less than 7 days were used for qPCR analysis. Quantitative PCRs with three technical replicates for each sample were performed in a CFX 96TM Real-Time System (Bio-Rad) in a total reaction volume of 10 μL consisting of 5 μL 2× Super Mix (Bio-Rad, 172-5201AP), 3 μL double-distilled water, 0.5 μL forward and reverse primer (10 μM each), and 1 μL cDNA template. The amplification efficiency in terms of highest *R*^2^ value was used to pre-optimize the amplification cycling parameters which included 95°C for 5 min followed by 40 cycles of 95°C for 20 s, 61°C for 20 s, and 72°C for 20 s. A melting curve analysis (MCA) was used to confirm specificity of the PCR products. In total, four genes coding for G protein subunit beta 1 (*Golf*), the regulator of G-protein signaling 2 (*RGS2*), G protein-coupled receptor kinase 2 (*GRK*), and *arrestin* from the olfactory transduction pathway were investigated. The 18S rRNA was used as a reference to calculate the relative expression levels of the genes investigated. All primers used in the present study were synthesized by Tsingke Biotech (Hangzhou, China). Sequence information of the primers is listed in Table 2.

**Table 2:** Primer sequences for the genes investigated and the internal reference 18S rRNA (F and R after the dash line in the primer name indicate forward and reverse primers, respectively).

Primers	Sequence (5′ to 3′)	Accession no.
*18S*-F	GCCAAGTAGCATATGCTTGTCT	GU017319
*18S*-R	AGACTTGCCTCCAATGGATCC	
*Golf*-F	GTCGGAGCATTATTCATTCA	MH370475
*Golf*-R	GGTAGCGTTGGAGATAGAG	
*RGS2*-F	CCTCAAGTCCGAGTTCTG	MH294433
*RGS2*-R	CTCTGGATGATGGCATTCT	
*GRK*-F	GTCGGTACTCATGGTTACA	MH155243
*GRK*-R	GGTCTTGTGCTGTCTGAA	
*Arrestin*-F	TCTACATCTCCACCTTCCA	MH141554
*Arrestin*-R	CCTCTTGTGAATCTTCTCTTC	


### Statistical Analysis

The effects of OA on the VCL, LIN, WOB approach to food source, the time taken for individuals to leave the acclimation area (latency time) and reach the food (response time), and the *in vivo* contents of neurotransmitters of black sea bream were analyzed using a linear mixed effects model with the treatment tank as a random variable using “R” statistical package lme4 (Bates, 2010; Bates et al., 2015). Expression levels of the genes investigated were compared to that of the control with the Duncan multiple range tests (Tallarida and Murray, 1987) using SPSS19.0. A *p*-value less than 0.05 was considered a statistically significant difference for all the statistical analyses.

## Results

### Effect of Ocean Acidification on Foraging Behavior

**FIGURE 1:**
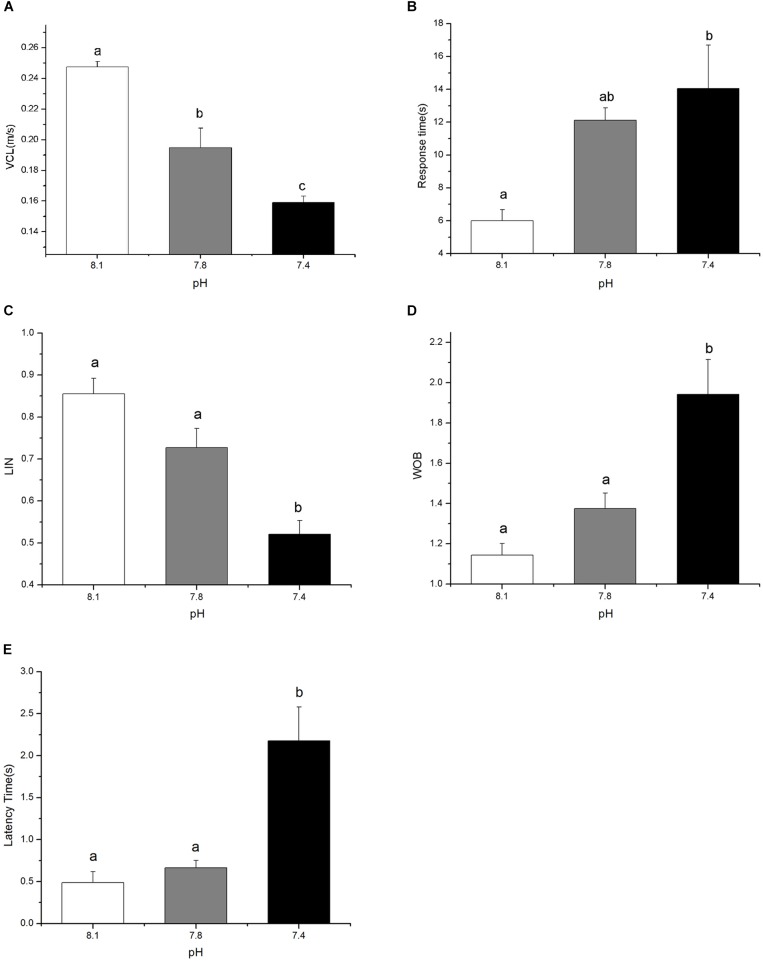
The curvilinear velocity (VCL, **A**), the response time (time it took for individuals to reach the food source, **B**), the linearity (LIN) of swimming path **(C)**, the wobble (WOB) of swimming path **(D)**, and the latency time (time it took for individuals to leave the acclimated area, **E**) of black sea breams after 15 days exposure to the control or *p*CO_2_ acidified seawater at pH 8.1, 7.8, and 7.4, respectively. All data were presented as the means ± SEM and groups not sharing the same superscripts are significantly different from each other at *p* < 0.05.

Exposure to elevated *p*CO_2_ for 15 days exerted a significant negative impact on the foraging behavior of black sea breams by reducing VCL and LIN whereas increasing latency time, response time, and WOB (Figure 1, *p* < 0.05). Black sea breams raised in acidified seawater approached food pellets at significantly slower speeds, with reduction of 21.32% at pH 7.8 and 35.72% at pH 7.4, respectively, compared to that of the control (Figure 1A, *F*_(2,8)_ = 54.1714, *p* = 0.0007). Though no significant difference was detected for treatment groups at pH 7.8, the time taken for individuals to reach the food source and leave the acclimated area were significantly increased for sea breams in pH 7.4, which were approximately 2.34 and 3.46 times higher than that of the control (Figures 1B,E, *F*_(2,8)_ = 9.03568, *p* = 0.0299 and *F*_(2,8)_ = 63.51385, *p* = 0.0005), respectively. Similarly, though no difference was observed for pH 7.8 treatment group, treatment in pH 7.4 led to significant changes in LIN and WOB of the individuals, with a decrease of 39.12% and an increase of 69.94% of the control (Figures 1C,D, *F*_(2,8)_ = 31.1332, *p* = 0.0025 and *F*_(2,8)_ = 19.4920, *p* = 0.0069), respectively.

### Effect of Ocean Acidification on the *in vivo* Contents of GABA and ACh

**FIGURE 2:**
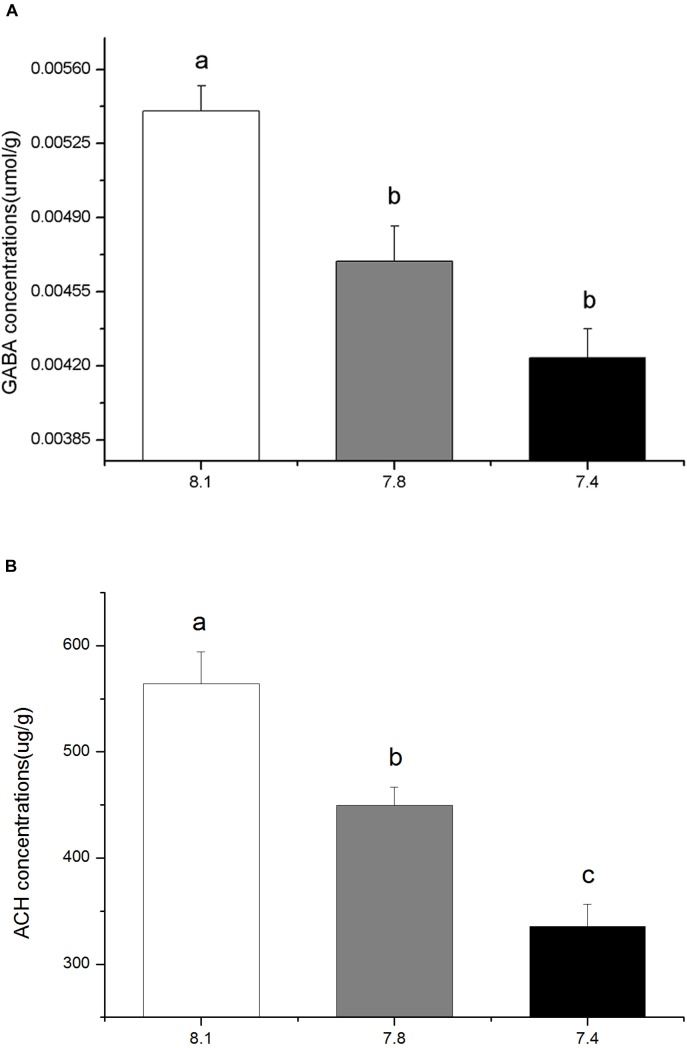
The *in vivo* contents of GABA **(A)** and the *in vivo* contents of ACh **(B)** in black sea breams exposed to 15 days of control or *p*CO_2_ acidified seawater at pH 8.1, 7.8, and 7.4, respectively. All data were presented as the means ± SEM and groups not sharing the same superscripts are significantly different from each other at *p* < 0.05.

The *in vivo* contents of GABA and ACh in the brains of black sea breams were significantly reduced after 15 days exposure to elevated *p*CO_2_. Compared to that of the control, the GABA contents in individuals in *p*CO_2_ acidified seawater declined by approximately 21.55% at pH 7.4 and 13.14% at pH 7.8 (Figure 2A, *F*_(8,26)_ = 14.4347, *p* = 0.0126). Similarly, the ACh contents for treatment groups at pH 7.4 and 7.8 were approximately 40.54 and 20.32% of that of the control, respectively (Figure 2B, *F*_(8,26)_ = 15.4873, *p* = 0.0110).

### Effects of Ocean Acidification on the Expressions of Genes From the Olfactory Transduction Pathway

**FIGURE 3:**
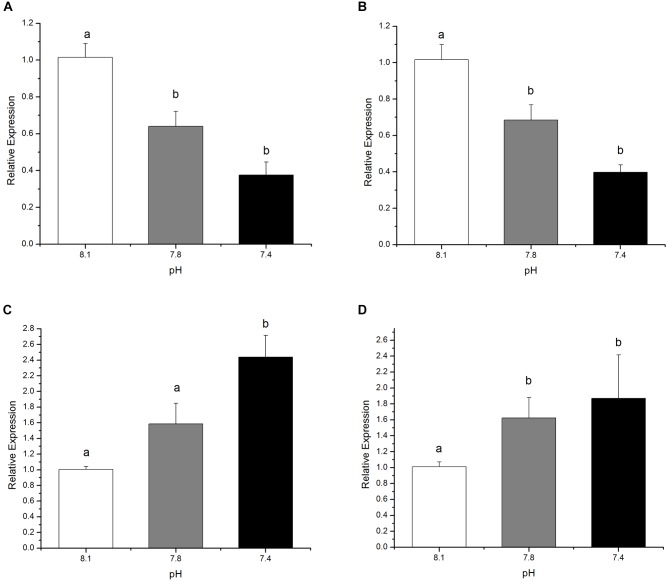
The expression of genes encoding *RGS2* **(A)**, *Golf* **(B)** *Arrestin* **(C)**, and *GRK* **(D)**, after 15 days exposure to the control or *p*CO_2_ acidified seawater at pH 8.1, 7.8, and 7.4, respectively. All data were presented as the means ± SEM and groups not sharing the same superscripts are significantly different from each other at *p* < 0.05.

The relative expressions of genes under investigation from the olfactory transduction pathway were significantly (*p* < 0.05) altered in black sea breams after 15 days exposure to acidified seawater. Compared to the control, a significant down-regulation of the genes encoding *Golf* and *RGS2*, which positively regulate olfactory transduction, was detected in the *p*CO_2_ elevated experimental groups. The relative expressions of genes encoding *RGS2* declined by 36.92% at pH 7.8 and 63.04% at pH 7.4 (Figure 3A, *F*_(8,26)_ = 32.85369, *p* = 0.0023), respectively, whereas *Golf* expression decreased by approximately 32.62% and 60.77% at pH 7.8 and 7.4, respectively (Figure 3B, *F*_(8,26)_ = 33.18895, *p* = 0.0022). In contrast, the expression of the genes encoding *GRK* and *arrestin*, two negative modulators for olfactory transduction, was significantly induced in individuals raised in acidified seawater, except for the expression of the genes encoding *arrestin* at pH 7.8. Specifically, the relative expressions of *arrestin* for treatment groups at pH 7.8 and 7.4 increased by circa 58.03 and 142.93%, respectively (Figure 3C, *F*_(8,26)_ = 22.13905, *p* = 0.0053). Expression of *GRK* increased by roughly 60.78% for pH 7.8 treatment group and 85.11% for pH 7.4 treatment group (Figure 3D, *F*_(8,26)_ = 2.67412, *p* = 0.1609).

## Discussion

Results obtained in the present study revealed that under future OA scenarios, black sea bream may swim in a more zigzag manner at a lower velocity and take a longer time locating food sources. Though inconsistent results have been reported in juvenile anemone fish, *Amphiprion melanopus* (Nowicki et al., 2012), our findings are comparable to most previous reports conducted in a range of other fish species. For instance, it took approximately 4 times longer for the shark *Heterodontus portusjacksoni* reared under elevated *p*CO_2_ at ∼1000 μatm for approximately 36 days in mesocosms to locate their prey through olfaction, as compared to controls (Pistevos et al., 2015). Similarly, it was shown that several days exposure to acidified seawater (*p*CO_2_ at 600 μatm) significantly altered the feeding behavior of the brown dottyback, *Pseudochromis fuscus*. This may result from olfactory impairment since the brown dottyback, after exposure to elevated *p*CO_2_ (600 and 950 μatm), spent approximately 20% less time in a water stream containing prey odor compared with controls (Cripps et al., 2011). Consequently, the impairment in foraging efficiency detected in the present study may leave black sea breams more vulnerable to malnutrition under future OA scenarios, especially when the potential food species may also suffer effects from similar high partial pressure of CO_2_ environment (Comeau et al., 2009; Saderne and Wahl, 2012).

Olfactory transduction is crucial for triggering behavioral responses, such as foraging, to odor cues (Arvedlund and Takemura, 2006). Within the compact cilia of the olfactory receptor neurons (ORNs), a cascade of enzymatic activity transduces the binding of an odorant molecule to a receptor into an electrical signal that can be transmitted to the brain (Kaupp, 2010). This process is initiated by the binding of an odorant to the olfactory receptor (*OR*), which activates the olfaction-specific G protein (*Golf*) and subsequently the adenylyl cyclase type III (*ACIII*), the olfactory cyclic nucleotide-gated channel (*CNGC*; composed of one B1b, one A4 and two A2 subunits), and the Ca^2+^-activated Cl^-^channel (*CaCC*) through a cascade reaction. Activation of these channels elicits the influx of Ca^2+^, Na^+^ and the exodus of Cl^-^ thereby generating an electrical signal, and the depolarization of the plasma membrane. This process is negatively regulated through inhibition of the *OR* by the phosphorylation of the *GRK*, *arrestin*, and protein kinase A (*PKA*), which activates ion exchangers and initiates the release of Ca^2+^ and K^+^ and the influx of Na^+^. In addition, the G-protein signaling 2 (*RGS2*) positively modulates olfactory transduction in this process by inhibiting *ACIII* and subsequently down-regulating the *PKA*, an inhibitor for the *OR* (Firestein, 2001; Kato and Touhara, 2009; Kaupp, 2010; Peterlin et al., 2014). Therefore, in the present study, the results that acidification suppressed the expression of positive regulators (*Golf* and *RGS2*) while inducing negative regulators (*GRK* and *arrestin*) in the olfactory transduction pathway, indicate a significantly hampered olfactory transduction in response to elevated *p*CO_2_.

**FIGURE 4:**
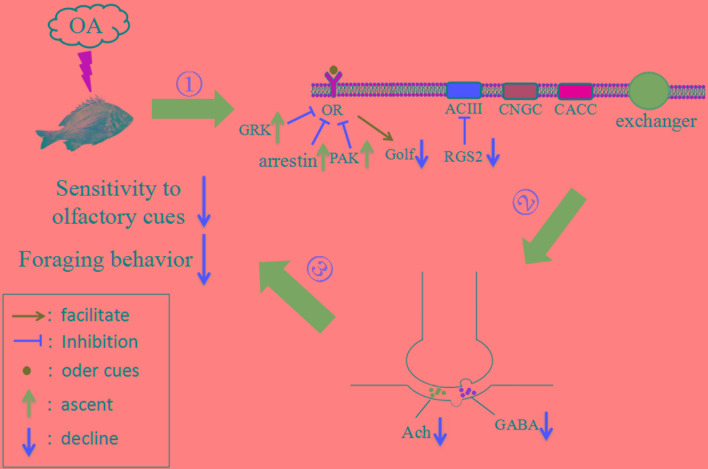
Working model summarizing tentative mechanistic pathway for impaired foraging behavior of black sea bream under future ocean acidification (OA) conditions, inferred by current study findings. (1) Exposure of black sea breams to elevated *p*CO_2_ suppresses the expression of positive regulators (*Golf* and *RGS2*) while induces negative ones (*GRK* and *arrestin*) in the olfactory transduction pathway and therefore hampers the transduction of olfactory signal. (2) OA leads to a significant reduction in the *in vivo* contents of GABA and ACh which interferes the transduction of neural signal. (3) Both (1) and (2) makes the fish raised in *p*CO_2_ acidified seawater less sensitive to the olfactory cues of food and therefore results in an impaired foraging behavior.

Responding to a threshold graded electrical potential, such as that generated by the olfactory transduction pathway, neurotransmitters are released into the synaptic cleft, where they bind to corresponding receptors and subsequently pass on the information to neighboring target cells. Therefore, both the neurotransmitters and their receptors are crucial for regulating physiological and behavioral responses of an organism to environmental variations (Nilsson et al., 2012; Clements et al., 2017; Peng et al., 2017). Currently, behavioral and olfactory impairment under simulated OA is thought to be caused by alteration of GABA receptor modulation of internal acid-base homeostasis (Nilsson et al., 2012; Chivers et al., 2014; Clements and Hunt, 2015; Regan et al., 2016; Schunter et al., 2016, 2018), leaving the potential impacts of neurotransmitter content largely overlooked. In addition, though it has been shown that upon stimuli, both GABA and ACh will be released into mitral cells, where nerve cells located on the olfactory bulb receive information from the olfactory receptor, indicating essential roles of both GABA and ACh in transmitting olfactory neural signals (Elaagouby and Gervais, 1992; Liu et al., 2013; Borin et al., 2014; Tatti et al., 2014), little is known about the response of ACh to elevated *p*CO_2_. Therefore, the significant reduction in the *in vivo* contents of both GABA and ACh under elevated *p*CO_2_ detected in the present study not only suggested that ACh along with GABA may participate in the regulation of OA induced behavioral changes, but also indicated a significant interference in the olfactory neural signal transduction pathway in black sea breams under near-future OA scenarios (Figure 4). In addition, the binding of signal molecules to corresponding receptors could also be affected by OA (Roggatz et al., 2016), which may also contribute to the hampered olfactory signal transduction detected. However, this inference requires further experimental confirmation.

In recent years, increasing evidence showed that some marine species can acclimate to high levels of CO_2_ over time (Form and Riebesell, 2012; Miller et al., 2012; Parker et al., 2012; Dupont et al., 2013; Zhao et al., 2016, 2018). Currently there is still no evidence for within- or *trans*-generational acclimation of behavioral responses to OA in fishes, possibly due to limited plasticity of functioning neurotransmitters (Munday, 2014; Welch et al., 2014). Currently, it remains unclear whether and to what extent fishes will adapt to ocean acidification, however, preliminary research showing considerable individual variation in the expression of CO_2_-sensitive genes in fishes indicates there may be scope (Schunter et al., 2016, 2018).

In conclusion, we show that the foraging behavior of black sea breams was significantly impaired by exposure to elevated *p*CO_2_ levels in this study, which may result from a reduced sensitivity to olfactory cues due to interference in the transduction of olfactory neural signals. To the best of our knowledge, this is the first study demonstrating the physiological mechanism with respect to the olfactory signal transduction underlying the high CO_2_ induced olfactory impairment. The findings of the present study together with currently available data suggest the mechanism underlying CO_2_-induced behavioral impairment could be consequences of multi-physiological changes and therefore should be examined and interpreted comprehensively. The impaired foraging efficiency detected in the current study may have considerable implications for wild populations and therefore for fisheries and resource managers.

## Author Contributions

RJ, HM, WH, and LG contributed conception and design of the experimental plan. RJ, SuW, GX, ShW, and ZS performed the experiments. RJ, HM, and LG performed the statistical analysis and wrote the manuscript.

## Conflict of Interest Statement

HM was employed by company Lucta (Guangzhou) Flavors Co., Ltd. The remaining authors declare that the research was conducted in the absence of any commercial or financial relationships that could be construed as a potential conflict of interest.
